# Emergence of New Norovirus Variants on Spring Cruise Ships and Prediction of Winter Epidemics

**DOI:** 10.3201/eid1402.061567

**Published:** 2008-02

**Authors:** Linda Verhoef, Evelyn Depoortere, Ingeborg Boxman, Erwin Duizer, Yvonne van Duynhoven, John Harris, Christina Johnsen, Annelies Kroneman, Soizick Le Guyader, Wilina Lim, Leena Maunula, Hege Meldal, Rod Ratcliff, Gábor Reuter, Eckart Schreier, Joukje Siebenga, Kirsti Vainio, Carmen Varela, Harry Vennema, Marion Koopmans, on behalf of the Food Borne Viruses in Europe Network

**Affiliations:** * Center for Infectious Disease Control, Bilthoven, the Netherlands; † European Centre for Disease Prevention and Control, Stockholm, Sweden; ‡ Food and Consumer Product Safety Authority, Zutphen, the Netherlands; § Health Protection Agency, London, England; ¶ Statens Serum Institut, Copenhagen, Denmark; # Institut Français pour la Recherche et l'Exploitation de la Mer, Nantes, France; ** Public Health Laboratory Centre, Hong Kong Special Administrative Region, People’s Republic of China; †† University of Helsinki, Helsinki, Finland; ‡‡ Norwegian Institute of Public Health, Oslo, Norway; §§ Institute of Medical and Veterinary Science, Adelaide, South Australia, Australia; ¶¶ Baranya County Institute of State Public Health Service, Pécs, Hungary; ## Robert Koch Institute, Berlin, Germany; *** Instituto de Salud Carlos III, Madrid, Spain

**Keywords:** Norovirus, epidemiology, virology, outbreaks, transmission, gastroenteritis, communicable diseases, surveillance, ships, research

## Abstract

In June 2006, reported outbreaks of norovirus on cruise ships suddenly increased; 43 outbreaks occurred on 13 vessels. All outbreaks investigated manifested person-to-person transmission. Detection of a point source was impossible because of limited investigation of initial outbreaks and data sharing. The most probable explanation for these outbreaks is increased norovirus activity in the community, which coincided with the emergence of 2 new GGII.4 variant strains in Europe and the Pacific. As in 2002, a new GGII.4 variant detected in the spring and summer corresponded with high norovirus activity in the subsequent winter. Because outbreaks on cruise ships are likely to occur when new variants circulate, an active reporting system could function as an early warning system. Internationally accepted guidelines are needed for reporting, investigating, and controlling norovirus illness on cruise ships in Europe.

## Abstract 2

A reporting system could provide early warning.

Norovirus is a highly infectious causal agent of a usually mild and self-limiting acute gastroenteritis. The symptoms of vomiting and diarrhea occur after a short incubation period of 8 to 72 hours. Although norovirus can cause sporadic cases (*1*), this contagious virus is often described as a cause of outbreaks (*2*–*5*). In Europe, norovirus outbreaks are reported to the Food Borne Viruses in Europe (FBVE) network. This network maintains a Web-based surveillance database containing data reported by 13 European countries (*6*).

In June 2006, the Dutch Food and Consumer Product Safety Authority (VWA) contacted the coordinator of the FBVE network, located at the National Institute for Public Health and the Environment (RIVM) in the Netherlands. The VWA had been notified of suspected norovirus outbreaks on 3 cruise ships operating in the Netherlands in the previous month. In the same week, ProMED reported a viral gastroenteritis outbreak on a Dutch-owned cruise ship operating out of the United Kingdom (*7*). Norovirus outbreaks on cruise ships are not normally reported to national surveillance centers in Europe, but having been alerted to these outbreaks, these centers recognized that the number of outbreaks was unusual. In Europe, norovirus outbreaks are highly seasonal, with most outbreaks reported from October through April (*8*,*9*). Further inquiries found that passengers on several ships sailing within European waters were experiencing outbreaks of gastroenteritis. This finding resulted in a coordinated investigation between the European Centre for Disease Prevention and Control (ECDC) and the FBVE network to identify or exclude a common source of infection (*10*,*11*). The investigation was based on the hypotheses that the possible rise in reported outbreaks was 1) reporting bias resulting from media attention and active investigation of these outbreaks, 2) an actual increase specific for cruise ships by means of a common source, or 3) a reflection of actual increased norovirus activity in the community. We describe the results of data collection at the European level by an international and multidisciplinary investigation team.

## Methods

Epidemiologic, virologic, and baseline data were collected from various sources. These sources included the FBVE network, ECDC, Food Safety Authorities, Early Warning Response System messages, diagnostic and reference laboratories, local health institutions, and ship owners.

### Definitions

A single outbreak was defined as a cluster of at least 3 people becoming ill within 3 days of each other with symptoms of acute gastroenteritis during 1 voyage with 1 group of passengers on board a ship. A ship-level outbreak was defined as successive single outbreaks occurring on 1 ship. An outbreak was confirmed if norovirus was detected in stool samples from >2 patients and was considered probable if norovirus was detected in only 1 patient’s sample or in >1 environmental samples. If descriptive clinical data suggested a viral cause but microbiologic proof for the causative agent was absent, the outbreak was considered as possibly caused by norovirus. Because norovirus outbreaks typically occur in winter, we defined a norovirus surveillance year as running from May through April of the next year to include a full winter season. Two periods were defined: off-seasonal, lasting from May through September; and seasonal, lasting from October through April of the following year.

### Data Collection

#### Epidemiologic Data

We included outbreaks that occurred on ships sailing within Europe and that were reported between January 1 and August 1, 2006. Information describing the outbreaks was collected; the dataset is given in a footnote of the Appendix Table. If an on-site outbreak investigation was performed, local authorities were asked to send their outbreak report to the investigation team.

#### Virologic Data

Environmental samples and patients’ stool samples were collected and were tested at different institutions by using local protocols, primarily reverse transcription–PCR (*12*). Virus information was collected to determine the causative agent and to determine whether identical strains indicated a common source for different ships. Sequences from a specific genomic region, the polymerase region A, were analyzed, which allowed an international comparison to be made (*13*). If this analysis could not be done at a local level, stool samples, RNA, or sequence information was sent to reference laboratories.

#### Background Data

The FBVE database (*6*) enables analyses of combined epidemiologic and virologic data. Baseline incidence was determined by analyzing the reported outbreaks, as registered in the FBVE database in March 2007. Outbreaks with onset from May 2002 through February 2007 were selected for the analyses. Because data from surveillance data collection were incomplete and associated with delays, to assess the number of outbreaks that occurred from May through June 2006, the FBVE network conducted an email survey within the network in July 2006. In addition, outbreak data for 2006 were obtained from Australia and Hong Kong and compared with data for 2005.

### Data Analysis

#### Epidemiologic Data

The following data were obtained retrospectively from outbreak reports: number and duration of outbreaks, overall attack rate, attack rates among passengers and crew, availability of (adequate) protocols and materials for cleaning, passenger flow during embarkation and disembarkation (possibility of contact between arriving and departing passengers), sick leave for crew, and policy for sick patients (isolation or not). For comparison of proportions, p values were calculated according to the χ^2^ test and Fisher exact test, if appropriate. Available epidemic curves were used to determine whether a point source infection was indicated through log-normal distribution (*14*), with patients clustering within 1.5× the incubation period range (CDC Manual, available from www.cdc.gov/health/botulism.htm; the manual was adapted for general epicurves, available from www.epi.state.nc.us/epi/gcdc/manual/Epicurves.pdf). Data were analyzed by using PEPI 4.0 (Programs for Epidemiologists; Sagebrush Press, Salt Lake City, UT, USA).

#### Virologic Data

Nucleotide sequences were aligned through Bionumerics software version 4.6 (Bionumerics package, Applied Maths, Ghent, Belgium). These sequences were then compared with consensus sequences by using a publicly available, Web-based, quick-typing tool (available from www.rivm.nl/bnwww).

#### Background Data

The differences between the off-seasonal number of reported outbreaks in 2006 (*x*_1_) and the off-seasonal numbers of reported outbreaks in previous years (*x*_2–5_) were compared; the difference was significant according to the following equation:


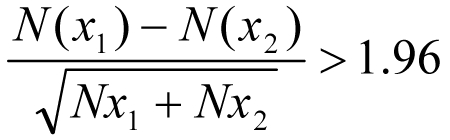


In addition, we compared the numbers of off-seasonal, seasonal, and cruise ship outbreaks in 2006 and 2007 with the numbers from previous years, from the FBVE database. Annual seasonal numbers of reported outbreaks were ranked to assess Spearman rank correlation coefficients. Poisson regression analysis was performed to determine whether the numbers of off-seasonal outbreaks were independent from the numbers of cruise-related outbreaks. In the FBVE database, outbreaks that occur on ships are reported in the category aircraft/ship/train/bus; analysis of cruise ship outbreaks used this category, which led to some misclassification of cruise ship outbreaks. Some additional information on setting is given in free text fields and, if available, was used to reduce misclassification. Data were analyzed by using SAS 9.1 for Windows (SAS Institute Inc., Cary, NC, USA).

## Results

During the study period, 43 single outbreaks were reported from 13 vessels: 14 (33%) of these were confirmed, 2 (5%) were considered probable, and 27 (63%) were considered possible norovirus outbreaks. For ship-level outbreaks, norovirus infection was confirmed for 10 (77%) vessels, 1 (8%) ship had probable norovirus infections, and 2 ships (15%) had possible norovirus infections.

### Epidemiologic Data

**Figure 1:**
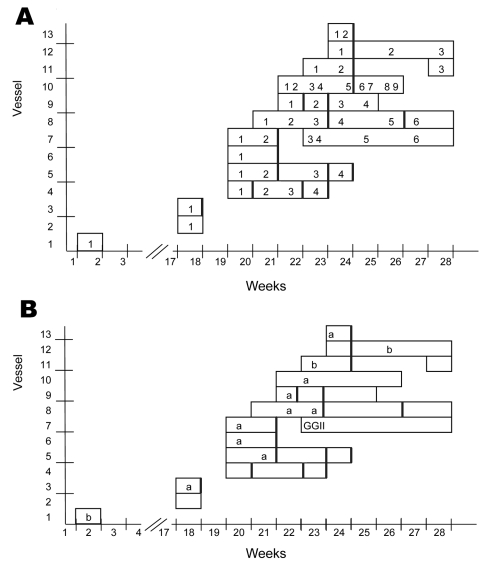
Number of outbreaks (A) and causative genotypes (B) for cruise-related outbreaks of norovirus for each ship from January through July 2006. Data were derived from multiple sources, active case finding, and case reports. Vertical black lines indicated markers for reported cleaning activities with extra intensity. a, GGII.4–2006a; b, GGII.4–2006b; GGII, GGII but variant unknown.

Of the 43 outbreaks, 1 occurred in January 2006; all others occurred from April 24 through July 21, with only a 2-week outbreak-free period (Figure 1). Three outbreaks on 3 ships occurred during the season; 40 outbreaks on 10 ships occurred during off-season months. The Appendix Table shows available epidemiologic and virologic data for each cruise ship that had confirmed or probable norovirus outbreaks. Overall attack rates varied from <1% to 41%. The highest attack rates were 48% for passengers and 19% for crew members. Ships 10 and 13, which were ferries, reported the lowest overall attack rates and higher attack rates for crew than for passengers (p = 0.021 for ship 10; p = 0.064 for ship 13).

#### Source of the Outbreaks

For 3 ships, the epidemic curve of the initial outbreak was available. One of these indicated a point source, which could not be identified during the outbreak investigation. For 7 of the 13 ships, at least 15 food suppliers were identified. Of these, 13 suppliers delivered products to 1 ship and 2 suppliers delivered to multiple ships Appendix Table. A common food source could not be identified for all ships. For 6 ships, no information on the food supplier was available.

A retrospective cohort study performed on 1 ship and case-control studies on 2 other ships could not find any evidence of a point source. Person-to-person spread was believed to be the predominant route of transmission as shown in these analytical studies and in another 6 descriptive reports.

#### Risk Factors for Multiple Outbreaks

Reports from local investigation teams were available for 7 of 9 ships that experienced multiple outbreaks and for 2 of 4 ships that experienced only 1 outbreak. These low numbers, including missing values, did not enable analysis to identify risk factors for multiple outbreaks. Descriptive information indicated the following risk factors: possible contact between boarding and disembarking passenger groups and cleaning with inappropriate materials for norovirus elimination during the first outbreak.

### Virologic Results

The norovirus sequences, detected in fecal or environmental samples, were all of the GGII.4 genotype but in 2 distinct new lineages, designated GGII.4–2006a and GGII.4–2006b Appendix Table and Figure 1) (*15*). Samples taken from 8 (73%) and 3 (27%) of 11 ships were identified with the GGII.4–2006a and GGII.4–2006b variant, respectively.

For 3 ships, the norovirus strains obtained from environmental samples were genetically identical to those obtained from patient samples. Positive environmental samples were derived from contact surfaces, which implied that person-to-person transmission through aerosols and contact with contaminated surfaces was possible. For 1 ship, samples of raspberries and tap water taken during the outbreak were found to be contaminated with norovirus. Whether the contamination was the source of the outbreak or resulted from contact with patients affected in the outbreak could not be determined.

### Analysis of Background Norovirus Activity

#### FBVE Database

**Figure 2:**
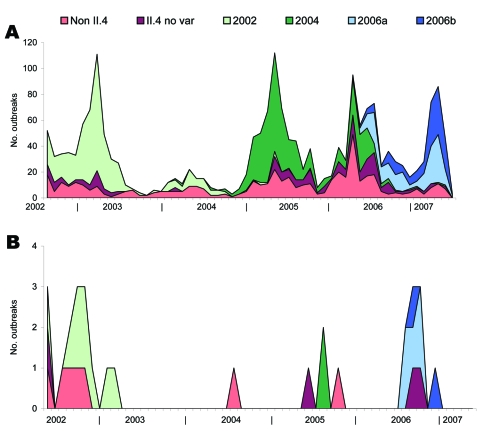
Cumulative outbreak data over time (2002–2007) from Food Borne Viruses in Europe network database. The total number of reported outbreaks (A) contrasted with the reported ship-related outbreaks (B). Both show norovirus strains involved.

From May 1, 2002, through February 28, 2007, a total of 9,425 norovirus outbreaks were reported to the FBVE network. A total of 2,480 outbreaks occurred during the norovirus surveillance year 2006–2007. For 8 of the countries, analysis of the number of off-season outbreaks from 2002 through 2006 was possible. A combined total of 137 outbreaks were reported by these countries during the 2006 off season. This number is higher than that for the same months in 2003 (n = 68, significant), 2004 (n = 127, not significant) and 2005 (n = 132, not significant) but lower than that for 2002 (n = 383, significant) when norovirus activity was very high. However, reporting for the year 2004–2005 has been considerably delayed (median 157 days, range 4–616). Since data were derived from the database on March 14, 2007, the numbers of reported outbreaks in the surveillance database are still increasing. An average of 5 aircraft/ship/train/bus-related outbreaks per year is reported in the FBVE database (Figure 2). The Spearman rank correlation coefficient was significant between the number of outbreaks in this category and the number of off-season outbreaks (R = 0.97; p = 0.0048). Poisson regression analysis showed that the annual number of off-season cruise ship outbreaks associated strongly with the total annual numbers of outbreaks in the subsequent season (p = 0.0078) as well as the same off-season (p<0.001).

In the norovirus surveillance year 2005–2006, GGII.4 strains were the predominant type identified (79%). The 2 new lineages within the GGII.4 genotype were first detected between January and March and displaced the resident GGII.4–2004 strains (Figure 2).

#### Survey

Of 13 collaborating countries in the FBVE network, 11 reported higher norovirus activity at the time of the ongoing cruise ship outbreaks. Australia and Hong Kong (*16*) experienced higher norovirus activity from January through June 2006, compared to the same period in 2005: a more than 10-fold increase was reported in Adelaide (147 for 2006, 117 of which occurred out of season from January through July), and Hong Kong reported 99 outbreaks from January through June 2006 compared with 46 outbreaks in 2005. In Australia and Hong Kong, outbreaks were associated with the new lineages 2006a and 2006b, respectively.

## Discussion

An unexpectedly high number of outbreaks on cruise ships in European waters in the spring and summer of 2006 triggered this investigation. Concomitant with this increase of norovirus outbreaks on cruise ships, we noted a marked increase in norovirus activity in the general population. The overall increase in norovirus activity in summer 2006 coincided with the emergence of 2 new norovirus GGII.4 strains and was followed by a higher number of outbreaks than usual in winter in 2006–2007 (*17*). Reporting of cruise ship related norovirus outbreaks may have been influenced by heightened attention from the media; however, the increase in reported land-based outbreaks is indicative of a real increase.

A similar situation occurred in the spring and summer of 2002, when a new variant of the GGII.4 strain emerged globally. This variant was found on cruise ships through the US Vessel Sanitation Program and in nursing homes and hospitals through the FBVE network (*18*–*20*). Retrospective analysis of 5 years of surveillance data from the FBVE network also showed a correlation between the number of off-season outbreaks on cruise ships and higher norovirus activity in the subsequent winter season. This recurring situation implies that cruises are possibly an early indicator for increased norovirus activity in the community because they are highly susceptible to norovirus outbreaks and mostly sail during warmer months of the year. A prospective and active surveillance program could demonstrate the validity of cruise ship outbreak incidence as a predictor of norovirus activity for the next season. After the first infection is introduced in this closed setting, an outbreak is likely to occur through person-to-person transmission (*9*). With the regular changing of passenger groups, the noroviruses on board are able to repeatedly infect a new susceptible population (*21*). Exhaustive control measures may not always be sufficient to eliminate the virus; a striking example is a positive environmental swab from a handle of a hand sanitation container, which was used before entering a restaurant. To get a better understanding of the epidemiology of noroviruses aboard cruise ships, we need a definition for a single outbreak that is more stringent than the one we used. That will only be possible with some level of routine monitoring of illnesses.

Our results could neither indicate nor exclude a point source or a common link through food or water. Information on food supply was incomplete and difficult to obtain. Separation of a potential point source from person to person or environmental transmission can optimally be investigated during a ship’s initial outbreak. This investigation was only conducted on 1 ship. That 2 different lineages of GGII.4 norovirus were involved provided some evidence that a common source for all ships was unlikely (Figure 1). That a common source was unlikely was further supported by the background data showing the emergence of the same viruses coinciding with increased reporting of outbreaks from all kinds of settings across the network (Figure 2).

Attack rates for crew and passengers differed. Attack rates for the crew were mostly lower than rates for passengers, which may have been due to short-term immunity, possibly acquired during successive outbreaks over long periods (*22*,*23*). However, reporting bias is possible because crew members may be reluctant to admit to being ill (*24*). The only 2 ships in which attack rates were higher for the crew were the 2 ferries. This finding is likely due to an underestimation of number of ill passengers, because their stay on board is shorter than the average incubation period. This explanation is supported by the fact that some ferry passengers were coincidentally discovered to have been ill during their return trip 2 days later.

Patient samples are needed to confirm the causative agent of the gastroenteritis outbreaks and to analyze the genetic sequence of viruses. Typing the norovirus strain will help show whether the outbreak is likely the result of reintroduction of the virus through a person. Person-to-person transmission is likely when community norovirus prevalence is high (*25*) and is a situation that shipping companies may be unable to prevent even if they are adhering to good cleaning procedures. Unfortunately, reintroduction of the virus through a new strain could not be determined in our study for 2 reasons. First, patient sampling when passengers fall ill on cruise ships in Europe is not standardized. Virus genotyping data for subsequent outbreaks were acquired from only 1 ship, where they were identical. Second, our data may have been insufficient to discriminate between GGII.4 variants and to determine sequence diversity. New variant strains of GGII.4 emerged over a vast geographic region within a short period of time, resulting in the finding of similar or identical sequences in outbreak strains collected throughout Europe. The level of genome analysis needed to enable discrimination between individual outbreak strains remains to be determined (*26*).

International outbreak surveillance can 1) provide background data on baseline activity of the virus and circulating strains and 2) facilitate tracing of foodborne sources, especially in the case of diffuse outbreaks that may result from centralized production and wide geographic distribution of products. At times of unusual numbers of outbreaks, additional active data collection helps compensate for underreporting, reporting delays, and helps elucidate export routes of foods. In the situation described here, thorough outbreak investigation was complicated as a result of continuation of trips through different countries during the course of outbreaks. This problem is a point of concern during potential common-source outbreaks, in which early detection of the source is crucial; this matter was considered by the ECDC, which launched an initiative for measures in Europe (*27*).

Gastroenteritis outbreaks on cruise ships may seem a luxury problem. However, at times of increased norovirus activity they are likely to occur and to receive much media attention. Higher norovirus activity appeared to coincide with emergence of new variant GGII.4 strains and with a higher number of cruise-related outbreaks in the preceding spring and summer. Cruise ship holidays create an environment in which norovirus is easily spread and outbreaks readily occur. Therefore, a reporting system for cruise ship–related outbreaks of gastroenteritis including virus detection and typing may function as an early warning system for high-epidemic winters. Such a system may enable a quick response and minimize negative effects of increased norovirus activity.

## Supplementary Material

Appendix Table Characteristics of norovirus outbreaks of gastroenteritis on vessels sailing within Europe, reported January 1–August 1, 2006*
