# Supplementary material for: Emergence of New Norovirus Variants on Spring Cruise Ships and Prediction of Winter Epidemics
Source: Emerg Infect Dis. 2008 Feb;14(2):238–43. doi: 10.3201/eid1402.061567 (PMC2600213; doi:10.3201/eid1402.061567)
Supplement: Appendix Table — Characteristics of norovirus outbreaks of gastroenteritis on vessels sailing within Europe, reported January 1–August 1, 2006* [file 06-1567_appT-s1.pdf]

Appendix Table. Characteristics of norovirus outbreaks of gastroenteritis on vessels sailing within Europe, reported January 1–August 1, 2006*

Ship no.†	Owner	Food supply	Point source first outbreak	Outbreak no.	Duration, d	Outbreak definition	Completeness, %‡	On-site investigation	Attack rate total		Attack rate passengers		No. + patient samples (no. taken)	Sequence patient sample	No. + env. samples (no. taken)	Sequenceenv. sample
									No. at risk	No. at-risk ill (%)	No. at risk	No. at-risk ill (%)				
1§	*A*	–	–		–			*No*	–	–	–	–		*GGII.4–2006b*	–	–
				1	–	Conf.	36	No	–	–	–	–	3 (5)	GGII.4–2006b	–	–
3§	*B*	–	–		–			*Yes*	*140*	*15 (11)*	–	–	*0 (1)*	–	*2 (4)*	*GGII.4–2006a*
				1	–	Prob.	79	Yes	140	15 (11)	–	–	0 (1)	–	2 (4)	GGII.4–2006a
5§	*E*	*X, others*	*No*		*30*			*Yes*	*2,590*	*104 (4)*	–	–	*6 (7)*	*GGII.4–2006a*	–	–
				2	9	Conf.	82	Yes	659	69 (10)	465	69 (5)	4 (4)	GGII.4–2006a	–	–
				4	2	Conf.	75	Yes	636	3 (<1)	450	3 (<1)	2 (3)	–	–	–
6§	*D*	*Y*	*No*		–			*Yes*	*166*	*61 (37)*	–	–	*4 (5)*	*GGII.4–2006a‡*	*3 (3)*	*GGII.4–2006a‡*
				1	–	Conf.	96	Yes	166	61 (37)	132	57(43)	4 (5)	GGII.4–2006a	3 (3)	GGII.4–2006a
7§	*F*	–	–		*>22*			*Yes*	*4,042*	*342 (8)*	–	–	*^{3}2 (17)*	*GGII.4–2006a*	*0 (0)*	–
				1	7	Prob.	79	Yes	725	108 (15)	504	90 (18)	^{3}1 (2)	GGII.4–2006a§	–	–
				4	–	Conf.	57	No	700	39 (6)	500	38 (8)	^{3}1 (10)	GGII	–	–
8§	*C*	*X, others*	–		*>29*			*Yes*	*3,578*	*293 (8)*	–	–	*15 (17)*	*GGII.4–2006a*	*0 (0)*	–
				2	6	Conf.	68	Yes	575	7 (1)	–	–	6 (6)	GGII.4–2006a¶	–	–

				3	6	Conf.	79	Yes	593	120 (20)	402	117 (29)	6 (6)	GGII.4–2006a¶	–	–
				6	–	Conf.	57	Yes	600	5 (<1)	–	–	3 (5)	–	–	–
9§	*G*	–	–		–			*Yes*	*12,124*	*369 (3)*	–	–	*8 (11)*	*GGII.4–2006a*	*1 (^{3}1)*	–
				1	–	Conf.	75	Yes	3,124	234 (7)	2,261	220 (10)	8 (11)	GGII.4–2006a	1 (^{3}1)	–
10§‡‡	*H*	*Z*	–		*>3*			*Yes*	*10,013*	*40 (<1)*	–	–	*3 (3)*	*GGII.4–2006a*	*0 (3)*	–
				4	1	Conf.	71	Yes	1,139	6 (<1)	1011	3 (<1)	3 (3)	*GGII.4–2006a§*	–	–
11§	*I*	–	–		–			*No*	*2,217*	*68 (3)*	–	–	*8 (8)*	*GGII.4–2006b*	*0 (0)*	-
				1	–-	Conf.	54	No	350	34 (10)	–	–	8 (8)	GGII.4–2006b	-	-
12§	*D*	*Y*	–		*>30*			*Yes*	*454*	*137 (30)*	–	–	*6 (7)*	*GGII.4–2006b‡*	*4 (13)*	*GGII.4–2006b‡*
				2	9	Conf.	100	Yes	147	61 (41)	115	55 (48)	6 (7)	GGII.4–2006b	4 (10)	GGII.4–2006b
13§‡‡	*J*	*X, others*	–		*8*			*Yes*	*2,442*	*40 (2)*	*2,142*	*32 (1)*	*7 (10)*	*GGII.4–2006a‡*	*1 (10)*	*GGII.4–2006a‡*
				1	1	Conf.	100	Yes	1,150	33 (3)	1,000	25(3)	4 (5)	GGII.4–2006a¶	0 (5)	
				2	3	Conf.	89	Yes	1,292	7 (<1)	1,142	7(<1)	3 (5)	–	1 (5)	GGII.4–2006a¶
Total	10								37,766	1,469 (4)			^{3}58 (87)		11 (^{3}31)	

*env., environmental; –, no data; Conf., confirmed; Prob., probable.

†For ships 2 and 4, none of the outbreaks could be contributed to norovirus; these ships are excluded from this table.

‡Name of the vessel; owner; food suppliers; serial number of outbreak; start and end of cruise; no. of people at risk; onset of first and last illness; no of cases; no., outcome, and sequence of stool samples taken; diagnostic or reference laboratory; route; tour operator; reporting date; no. of crew and passengers at risk; no. of cases among crew and passengers; dates of stool samples; number, date, outcome and sequence of environmental samples.

§Single outbreak, a cluster of at least 3 people becoming ill within 3 d of each other with symptoms of acute gastroenteritis during 1 voyage with a single group of passengers on board a ship; ship-level outbreak, successive single outbreaks occurring on 1 ship (nos. in italics).

¶Identical sequences detected in environmental and patient samples.

#Several mutations compared with GGII.4–2006a in other outbreaks and identical for ships nos. 7 and 10.

**One mutation compared with GGII.4–2006a in other outbreaks and unique when compared with strains from other vessels.

††If exact numbers of cases or population at risk were missing, estimates obtained from the ship owners were used.

‡‡Ships 10 and 13 were ferries, resulting in a potential underestimation in the duration, size, and attack rate of the outbreaks.
